# Insulin improves memory and reduces chronic neuroinflammation in the hippocampus of young but not aged brains

**DOI:** 10.1186/s12974-015-0282-z

**Published:** 2015-04-02

**Authors:** Linda Adzovic, Ashley E Lynn, Heather M D’Angelo, Alexis M Crockett, Roxanne M Kaercher, Sarah E Royer, Sarah C Hopp, Gary L Wenk

**Affiliations:** Department of Psychology, Ohio State University, 1835 Neil Ave, Columbus, OH 43210 USA; Department of Neuroscience, Ohio State University, Columbus, OH 43210 USA

**Keywords:** Aging, LPS, Rat, Inflammation, Insulin, Memory

## Abstract

The role of insulin in the brain is still not completely understood. In the periphery, insulin can decrease inflammation induced by lipopolysaccharide (LPS); however, whether insulin can reduce inflammation within the brain is unknown. Experiments administrating intranasal insulin to young and aged adults have shown that insulin improves memory. In our animal model of chronic neuroinflammation, we administered insulin and/or LPS directly into the brain via the fourth ventricle for 4 weeks in young rats; we then analyzed their spatial memory and neuroinflammatory response. Additionally, we administered insulin or artificial cerebral spinal fluid (aCSF), in the same manner, to aged rats and then analyzed their spatial memory and neuroinflammatory response. Response to chronic neuroinflammation in young rats was analyzed in the presence or absence of insulin supplementation. Here, we show for the first time that insulin infused (i.c.v.) to young rats significantly attenuated the effects of LPS by decreasing the expression of neuroinflammatory markers in the hippocampus and by improving performance in the Morris water pool task. In young rats, insulin infusion alone significantly improved their performance as compared to all other groups. Unexpectedly, in aged rats, the responsiveness to insulin was completely absent, that is, spatial memory was still impaired suggesting that an age-dependent insulin resistance may contribute to the cognitive impairment observed in neurodegenerative diseases. Our data suggest a novel therapeutic effect of insulin on neuroinflammation in the young but not the aged brain.

## Introduction

Aging is characterized by chronic low-level neuroinflammation [1-4]. The consequences of neuroinflammation, associated with microglial activation, operating across a timescale of decades, are carefully regulated until, due to normal aging, there is a gradual shift to a non-equilibrium state that is permissive for neurodegenerative processes [5-7]. Vulnerable brain regions, particularly the hippocampus, are likely exposed for many decades to a complex and varying blend of microglia in alternative activation states [8-10]. Dysregulation of insulin signaling is also associated with many age-related neurodegenerative diseases that also present with chronic neuroinflammation and that may contribute to cognitive deficits [11-13]; however, the link between these two characteristics and their interaction in the neurodegenerative processes has not been fully investigated.

In the periphery, insulin can decrease the inflammatory response [14] by reducing the production of cytokines, such as interleukin-1β (IL-1β) and tumor necrosis factor alpha (TNFα), and by decreasing protein kinase C zeta (PKCζ) protein expression [15,16]. PKCζ protein was shown to play a role in the activation of nuclear factor kappa B (NF-κB) pathway [17] which is necessary to enhance the inflammatory response. Interestingly, insulin was reported to suppress the expression and activity of inducible nitric oxide synthase (iNOS) [18] and cyclooxygenase-2 (COX-2) [19] by diminishing the activation of the NF-κB pathway [20] with a consequent reduction of nitric oxide and prostaglandins. The pro-inflammatory cytokine IL-1β can phosphorylate the insulin receptor substrate protein at Serine 307 [21] which may then negatively or positively [22,23] regulate insulin signaling. It is unknown whether insulin exhibits a similar neuroprotective role in the brain. The epidemiological data suggest that brain insulin resistance flanks both cognitive decline and progressive neuroinflammation in neurodegenerative diseases [11]; in particular, type II diabetes represent a risk factor to develop Alzheimer’s disease [24-28].

The insulin receptor (IR) is abundant in neurons and is highly concentrated in the hippocampus, particularly around the synaptic area [29,30]. Insulin can reach the brain by passing through the blood brain barrier (BBB) and can also be synthesized by neurons and released upon membrane depolarization [31]. Insulin signaling plays an important role in neuronal survival [32] and in learning and memory [33]. Intranasal insulin administration improved memory in young human subjects [34-36] and in patients with cognitive deficits associated with mild-stage Alzheimer’s disease [11,37]. The molecular mechanism through which insulin improves memory is still under investigation; however, recently, it was shown that insulin phosphorylates AMPA receptor at Serine 831 that is associated with the early stage and maintenance of long-term potentiation [37].

In the current study, we analyzed the role of insulin in spatial memory and in the inflammatory process within the hippocampus for memory regulation. Several laboratories have used intranasal insulin administration to deliver insulin to the brain [34,35]; we infused insulin directly into the young rat brain, in the presence or absence of neuroinflammation, for 4 weeks; we used recombinant human insulin which was shown to activate the insulin signaling in murine models [37,38].

In addition to analyzing the role of insulin in young rats, we also analyzed the role of insulin in aged rats. We assessed spatial memory and compared the behavioral and biochemical consequences of insulin administration. We have previously chronically infused low-doses of lipopolysaccharide (LPS) into the fourth ventricle to selectively stimulate the Toll-like receptor-4 (TLR4) complex expressed by microglia [39,40]. Administered in this fashion, the overall dose of LPS is not sufficient to produce any peripheral manifestations of infectious disease processes (such as elevated serum levels of inflammatory cytokines) or downregulation in TLR4 gene expression in the brain [41-47].

Here, for the first time, we show that insulin decreased the inflammation in the hippocampus of young rats by reducing the neuroinflammatory response and by rescuing spatial memory. Furthermore, insulin improved spatial memory in young rats. In contrast, we report that the aged brain was unresponsive to the insulin infusion. These results offer insight into the potential role of age-dependent insulin resistance with the cognitive decline associated with Alzheimer’s disease.

## Materials and methods

### Subjects and study design

Two separate studies were conducted in parallel. The first study used 64 young (3 months old) male F-344 rats (Harlan Sprague-Dawley) rats that were divided into four groups: artificial cerebral spinal fluid (aCSF)-, aCSF + insulin-, LPS-, and LPS + insulin-infused (i.c.v.). The second study used 27 aged (21 months old) male F-344 rats (Harlan Sprague-Dawley) divided into two groups: aCSF- and i.c.v. Comparisons were made to each age group’s respective aCSF control. Aged rats were not infused with LPS for three reasons: first, we have previously shown [41] that when LPS was chronically infused into the fourth ventricle of young and aged rats, only the young rats demonstrated impaired spatial memory performance in the Morris water maze task; the performance of aged rats was not further impaired by chronic infusion with LPS. Second, chronic infusion of LPS into the fourth ventricle of old rats did not significantly increase the number of activated microglia [41]. Third, the intent of our study was to determine whether insulin infusion in the brain could reduce the naturally occurring age-associated neuroinflammation.

The rats were maintained on a 12/12-h light-dark cycle in a temperature-controlled room (22°C) with free access to food and water and with lights off at 09:00 AM. All rats were given health checks, handled upon arrival, and allowed at least 1 week to adapt to their new environment prior to surgery. All rats were sacrificed during the dark phase of the diurnal cycle. Body weight and blood samples, withdrawn from the saphenous vein, were acquired weekly for the glucose and weight change analysis. We certify that the experiments were carried out in accordance with the National Institute of Health Guide for the Care and Use of Laboratory Animals (NIH Publications No. 80-23) revised 1996. We also certify that the formal approval to conduct the experiments has been obtained from the Institutional Animal Care and Use Committees of The Ohio State University.

### Surgery

aCSF (140 mM NaCl, 3.0 mM KCl, 2.5 mM CaCl_2_, 1.0 mM MgCl_2_, and 1.2 mM Na_2_HPO_4_ adjusted to pH 7.4), LPS (0.25 μg/hr, 1 mg/ml prepared in aCSF; E. coli, serotype 055:B5, TCA extraction, Sigma-Aldrich, St. Louis, MO, USA) or insulin (5 mU per day, insulin human recombinant cat #I2643, Sigma-Aldrich, St. Louis, MO, USA) were chronically (4 weeks) infused via a double cannula that was implanted into the fourth ventricle (−2.5 mm AP and −7.0 mm DV relative to lambda) and attached (via Tygon tubing, 0.06 O.D., Saint Gobain, Valley Forge, PA, USA) to an osmotic minipump (Alzet model #2004, to deliver 0.25 μl/hr; Durect Corp., Cupertino, CA, USA) as previously described [41,46,48]. A dosage of 5 mU insulin per day was shown to have no effect on daily food intake or body weight [49]. All the rats were infused for 28 days until the tissue collection day. Post-operative care included lidocaine 1% solution applied to the exposed skin upon closure and 2 ml of isotonic saline by subcutaneous injection to prevent dehydration during recovery. The rats had 23 days post-surgery recovery before starting the behavioral task.

### Behavioral testing and sacrifice

Morris water maze task (MWM): the rats were handled daily for 5 days before behavioral testing began. Spatial learning ability was assessed for all rats using the MWM, with a 170 cm diameter water pool with gray walls. The water was maintained at room temperature (22°C). The pool was in the center of a room with multiple visual stimuli as distal and proximal cues. The clear, circular escape platform was 10 cm in diameter. For the spatial (hidden-platform) version of the water task, a circular escape platform was present in a constant location, submerged 2.5 cm below the water surface. The rats were tracked using Noldus Ethovision 3.1 tracking and analysis system (Noldus, Leesburg, VA,USA). Beginning 24 days after surgery, each rat performed 6 trials per day for four consecutive days (24 trials total), with a 60-min inter-trial interval. Each rat was released into the water on each trial from one of six locations spaced evenly around the pool, which changed so that the rats did not start from the same location twice in 1 day. After the rat found the escape platform or swam for a maximum of 60 s, it was allowed to remain on the platform for 30 s. At the end of the fourth day, the platform was removed and a standard probe trial was conducted. After the probe test, all rats were also tested with the platform raised 2 cm above the surface of the water in a location different from the previous trials in order to control deficits in visual acuity. The effects of normal aging, insulin, and/or inflammation in cognition were assessed by comparing their latency to find the platform.

The day after the completion of behavioral testing, each rat was deeply anesthetized with isoflurane. Rats used for histology were prepared for a transcardiac perfusion with cold saline containing 1 U/ml heparin, followed by 4% paraformaldehyde in 0.1 M phosphate buffer, pH 7.4. The brains were then post-fixed overnight in the same fixative and then stored (4°C) in phosphate buffer saline (PBS), pH 7.4. Rats used for biochemistry from each group were rapidly decapitated; their hippocampi were quickly dissected on ice. The left and right hippocampi were randomly chosen for either protein or gene expression analyses and stored (−80°C) until processed. Blood was collected during the rapid decapitation procedure. After centrifugation at 4°C for 15 min 2,500 × *g*, serum was collected and assayed.

#### Immunocytochemistry

The immunocytochemistry was performed as previously described [50]. The monoclonal antibody OX-6 (final dilution 1:200, Pharmigen, San Diego, CA, USA) was used to visualize activated microglial cells only. This antibody is directed against class II major histocompatibility complex (MHC II) antigen, an indicator of activation. The location of immunohistochemically defined cells was examined by light microscopy. Quantification of cell density in the reconstructed hippocampal coronal sections (at least five sections from each rat) was assessed with Nikon 80i documentation system with DS-5 M-L1 digital camera using Elements 3.1 (Nikon Instruments, Melville, NY, USA) and quantified using MetaMorph imaging software (Universal Image Corporation, West Chester, PA, USA).

### Protein assays

#### Cytokine array

Frozen (−80°C) hippocampi were placed in a BioPlex Cell Lysis solution (Bio-Rad, Richmond, CA, USA), and total proteins were extracted according to the manufacturer’s instructions. Hippocampal levels of TNFα, interleukin-1-alpha (IL-1α), IL-1β, and IL-6 were quantified simultaneously with a magnetic bead immunoassay (Bio-Rad, BioPlex Pro Rat Standard, 171-K1002M, Bio-Rad, Richmond, CA, USA). A mixture of distinct capture beads (fluorescently dyed microspheres) each with a specific spectral address and conjugated to an antibody against one of the cytokines listed above were quickly dispensed across a 96-well plate and protected from light. Samples and antigen standards were added in duplicate and incubated for 1 h at 700 RPM at RT; unbound materials were washed away (3×). Then biotinylated detection antibodies directed against each of the proteins were added for 30 min at 700 RPM at RT; unbound materials were washed away (3×). Each well was then incubated for 10 min at 700 RPM at RT with a reporter dye, streptavidin-phycoerythrin conjugate (SA-PE), which binds to the detection antibody; unbound materials were washed away (3×). Each well was then suspended in assay buffer and shaken at 1,100 RPM for 30 s. Plates were analyzed using a Bio-Plex MAGPIX multiplex reader (Bio-Rad, Richmond, CA, USA). Values were compared to standard curves. All of the biomarkers were well above the background detection level of the assay. Protein was quantified in brain homogenates using the Bradford protein assay (Bio-Rad, Richmond, CA, USA). The results are reported as picograms/ml protein.

#### Western blot analyses

The hippocampi were lysed and analyzed using Western blot as previously described [37]. The antibodies for protein kinase-B (AKT), p-Akt (Ser 473), p-Akt (Thr 308), insulin receptor substrate-1 (IRS-1), IGF1-R, and the secondary anti-rabbit IgG, HRP-linked were supplied by Cell Signaling Technology (Danvers, MA, USA); the PKCζ (C-20) antibody was supplied by Santa Cruz Biotechnology (Dallas, TX, USA); and the insulin receptor antibody was supplied from Abcam (Cambridge, UK). The images were acquired with the enhanced chemiluminescence detection system (Pierce, Waltham, MA, USA). The data were quantified with Image-J software from NIH.

### Reverse transcription polymerase chain reaction

Total RNAs was extracted from hippocampi using PureZol (Bio-Rad, Richmond, CA, USA) followed by clean-up with a NucleoSpin RNA II kit (Macherey-Nagel, Düren, Germany) according to the manufacturers’ instructions. The total RNA (1 μg) from each sample was reverse-transcribed using the iScript reverse transcription Supermix for RT-qPCR (Bio-Rad, Richmond, CA, USA) to generate a cDNA library. Primers (see Table 1) were designed for each gene using the PrimerQuest software (Integrated DNA Technologies, Coralville, IA, USA; Table 1). Primers and Sso Advanced SYBR Green Supermix (BioRad, Richmond, CA, USA) were prepared with RNase-free water. For PCR amplification, mix (19 μl) was added to RT reaction (1 μl) previously diluted (1:20). Assays were run in triplicate on the CFX96, C1000 Thermal Cycler (Bio-Rad, Richmond, CA, USA). Amplification conditions were as follows: 95°C for 30 s, 40 cycles of PCR (denaturation: 95°C for 5 s, annealing/extension: 60°C for 30 s), and melting curves. Two negative controls were performed during each quantitative PCR experiment: reaction without the reverse transcription to confirm the absence of genomic DNA contamination and samples with no added cDNA template to prove the absence of primer dimers. Glyceraldehyde-3-phosphate dehydrogenase (GAPDH) was used as a housekeeping gene. The cycle (Ct) at which expression levels crossed threshold was normalized to the Ct of the housekeeper GAPDH, producing ∆Ct with arbitrary units of total gene expression. All plates were evaluated with respect to -RT and H_2_O controls.

**Table 1:** **Primer sequences for gene expression analyses**

**Gene**	**Accession number**	**Primer sequences F: forward; R: reverse**	**Annealing temp (°C)**	**Product length**
IL1ß	NM_031512	F: ACCTGCTAGTGTGTGATGTTCCCA	59.9	109
		R: AGGTGGAGAGCTTTCAGCTCACAT	59.9	
TNFα	X66539.1	F: CTGGCCAATGGCATGGATCTCAAA	60	97
		R: AGCCTTGTCCCTTGAAGAGAACCT	60	
GADPH	NM_017008	F: TGACTCTACCCACGGCAAGTTCAA	59.9	141
		R: ACGACATACTCAGCACCAGCATCA	60	
CREB1	NM_134443	F: TGTTCAAGCTGCCTCTGGTGATGTA	60	141
		R: ACCTCTCTCTTTCGTGCTGCTTCTT	60	
BDNF	BC087634	F: AGCCTGTGTACTTTGTGTCCGAGA	60.3	123
		R: TGGACGTTTGCTTCTTTCATGGGC	60.3	
IR	NM_017071	F: AGACCTTCGAGGATTACCTGCACAAC	60.3	118
		R: TTGCCCACCTCTTCAAGGGATCTT	60.4	
PRKCζ	NM_022507	F:AGAGGGATCATCTACCGGGACCTAA	60.2	85
		R:ACCTCTCTCTTTCGTGCTGCTTCTT	60.1	
NGF β	NM_001277055	F: ACTTCCAGGCCCATGGTACAATCT	60.2	145
		R: ATGTCCGTGGCTGTGGTCTTATCT	60	
CAMKIIα	NM_012920	F: ACCAACACCACCATCGAGGATGAA	60.4	135
		R: TGTCATTCCAGGGTCGCACATCTT	60.6	
PSD95	NM_019621	F:AGCGACGAGAGTGGTCAAGGTTAAAG	60	139
		R:CCAAGGATGATGATGGGACGAGCATA	60	
SYN1	NM_019133	F: TCTCCAGCACACCTTGCTCCTAATC	60	128
		R:CTGGTTGGTGGATAGTAGGTGGCATTT	60	

BDNF, brain-derived neurotrophic factor; CaMKIIα, Ca2+/calmodulin-dependent protein kinase II-alpha; CREB1, cAMP response element-binding protein-1; GAPDH, glyceraldehyde-3-phosphate dehydrogenase; IL, interleukin; iNOS, inducible nitric oxide synthase; IR, insulin receptor; NGF β, nerve growth factor-beta; PRKCζ, protein kinase C-zeta; PSD95, postsynaptic density protein 95; SYN1, Synapsin I; TNF-α, tumor necrosis factor-alpha.

### Statistic analyses

Statistical analyses were conducted out using SPSS software (version 20). A general linear model, repeated measures analysis of variance (rANOVA) and Fisher’s protected least significant difference for *post hoc* multiple comparisons were used. The Fisher’s *post hoc* test was utilized only if the overall *F*-value was significant for the effect. *P* < 0.05 was considered statistically significant. Graphs were prepared using GraphPad Prism (GraphPad Software, La Jolla, CA, USA) and are shown with the standard error of the mean represented by error bars. At least five rats for each treatment were used to obtain the data for gene expression, cytokine essay, and Western blot detection.

## Results

### Behavior

**Figure 1:**
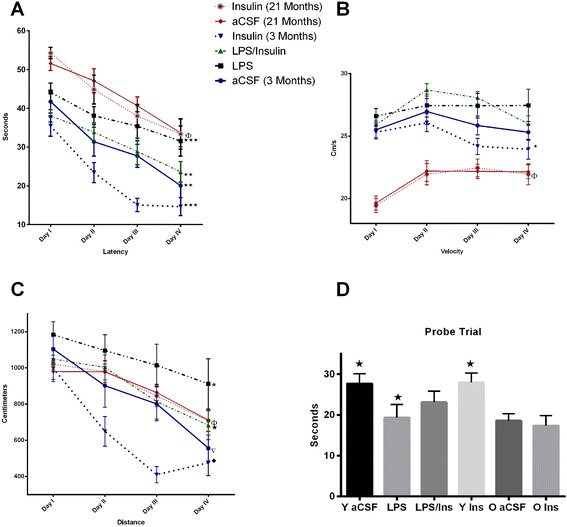
**Morris water maze performance. (A)** Latency: in the young groups, the performance was significantly impaired by LPS infusion; performance recovered when insulin was infused with LPS (see text). Surprisingly, the young rats receiving only insulin performed better compared to all other groups. Conversely, in the aged rats, insulin did not improve performance as compared to the controls. **(B)** Velocity: in the young rats, insulin significantly improved performance *versus* LPS and LPS/insulin groups (*P* < 0.05). **(C)** Distance: the distance to find the platform was significantly smaller in the insulin group (*P* < 0.05) over the 4 days while it was greater in the LPS group. Insulin/LPS was significant *versus* LPS (*P* < 0.05) and *versus* insulin (*P* < 0.01). Insulin treatment reduced the distance swam as compared to aCSF (*P* < 0.05). **(D)** Probe trial: the performance was impaired by LPS infusion as compared to aCSF and insulin (*P* < 0.05). (Stars) The aged rats showed no differences, *P* > 0.05, between aCSF and insulin-infused groups.

An rANOVA performed on the latency results (Figure 1A) revealed an overall main effect of treatment group (*F*_5,83_ = 13.2, *P* < 0.0001) and testing day (*F*_3,249_ = 52.5, *P* < 0.0001). *Post hoc* analyses determined that performance was significantly impaired by LPS infusion (*P* = 0.013) and the age of the rat (*P* < 0.001), as compared to young aCSF-infused rats. Insulin treatment significantly improved the performance in young rats, as compared to either aCSF-infused (*P* = 0.008) or LPS-infused (*P* = 0.028) young rats. Insulin treatment did not improve the performance of the aged rats (*P* > 0.05) as compared to aCSF-infused aged rats.

An rANOVA of swim speed (Figure 1B) revealed an overall main effect of treatment group (*F*_5,83_ = 18.8, *P* < 0.001). *Post hoc* analyses determined that swim speed was significantly impaired by the age of the rat (*P* < 0.001). Neither the LPS nor insulin infusions had significant effects on swim speed (*P* > 0.05). The analysis of variance of the distance swam to find the platform (Figure 1C) revealed an overall main effect of treatment group (*F*_5, 529_ = 16.1, *P* < 0.001). *Post hoc* analyses determined that performance was significantly impaired by LPS infusion (*P* < 0.001) but not by the age of the rat (*P* = 0.33). The insulin infusion significantly reduced the distance swam by the young rats, as compared to young rats that were infused with aCSF (*P* < 0.001). Insulin treatment did not significantly reduce the distance swam by the aged rats (*P* > 0.05).

### Reverse transcription PCR and Bioplex

**Figure 2:**
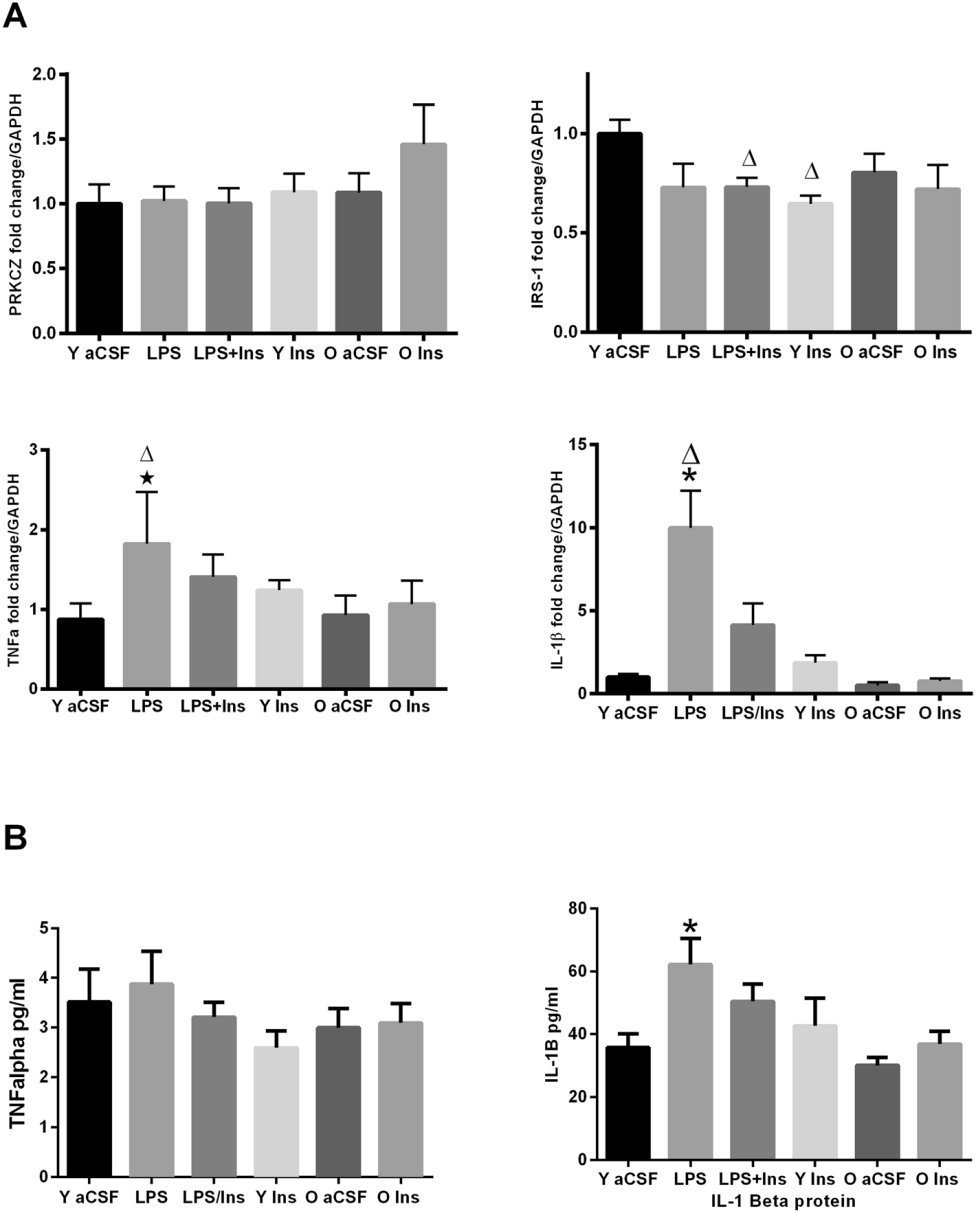
**RT-PCR and cytokine analyses. (A**) Gene expression: PRKCZ gene expression did not differ between groups, contrary to the protein level. IRS-1, top right graph, was decreased in the two young groups receiving insulin as compared to the vehicle group Δ (*P* < 0.05); in the young rats, IL-1β mRNA was increased in the LPS-infused group, *P < 0.01, *versus* aCSF and decreased, Δ*P* < 0.03 *versus* LPS + insulin. TNFα mRNA was increased by LPS infusion, *P < 0.01, *versus* aCSF and decreased, Δ*P* < 0.01, *versus* LPS + insulin. **(B)** No significant difference, *P* > 0.05, in the level of TNFα while IL-1β level was increased, *P < 0.01, as compared to aCSF. The aged rats showed no differences, *P* > 0.05, between aCSF and insulin-infused groups.

The gene expression, analyzed with general liner model univariate ANOVA between subjects (Figure 2), revealed an overall main effect of treatment group on IL-1β (*F*_3,27_ = 3.25, *P* < 0.001) and TNFα (*F*_3,20_ = 5.05, *P* = 0.007) gene expression and IL-1β protein levels (*F*_5,25_ = 4.51, *P* < 0.01). LPS infusion significantly increased IL-1β and TNFα gene expression (*P* < 0.01) and IL-1β (*P* < 0.01) protein levels. Insulin infusion significantly attenuated the effect of LPS on IL-1β and TNFα gene expression and on IL-1β protein level (*P* < 0.05) but did not decrease TNFα protein (*P* > 0.05). IRS-1 mRNA expression significantly decreased in young insulin and insulin + LPS treated group when compared to the vehicle (*P* < 0.05).

Aged rats showed no significant changes (*P* > 0.05) in IL-1β gene expression or protein levels. Gene expression of insulin receptor, insulin-like growth factor 1 receptor (IGF-1 R), cAMP response element-binding protein (CREB), brain-derived neurotrophic factor (BDNF), PRKCZ, NGFβ, Ca2+/calmodulin-dependent protein kinase II (CaMKII), postsynaptic density protein 95 (PSD95), and synapsin I (SYN1) were also unchanged.

### Western blot

**Figure 3:**
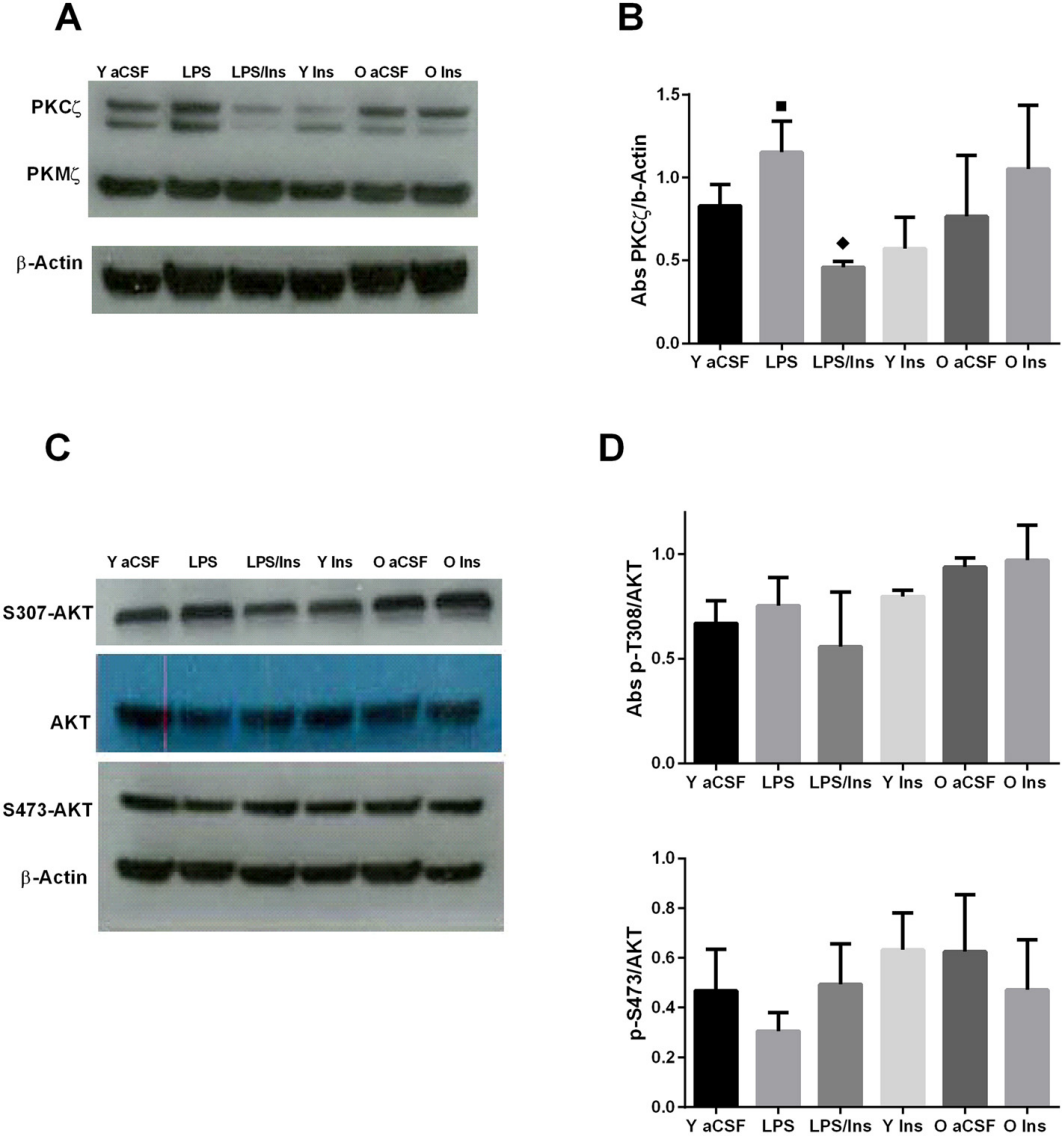
**Western blot analyses.** The infusion of LPS into the fourth ventricle increased the protein level **(A**, **B)** of PKCζ, black square, *P* < 0.05 *versus* aCSF. Insulin treatment reduced, black diamond, *P* < 0.001, PKCζ levels as compared to LPS. **(C**, **D)** No significant changes were observed for p-AKT Threonine 308 or Serine 473.

An rANOVA revealed an overall main effect of treatment group on hippocampal levels of PKCζ protein (*F*_5,14_ = 6.24, *P* = 0.003, Figure 3). LPS infusion increased PKCζ protein levels (*P* = 0.02) while insulin infusion significantly attenuated this effect (*P* < 0.001). Aged rats showed no significant changes (*P* > 0.05) in PKCζ protein expression. No significant (*P* > 0.05) changes in p-AKT Thr 308, p-Serine 473 AKT, or protein kinase M-zeta (PKMζ) protein levels were observed. In contrast with the results in young rats, insulin infusion into the brains of aged rats did not change PKCζ protein levels (*P* > 0.05) or induced any change in AKT protein expression or phosphorylation. Protein levels of CREB, BDNF, CAMKII, IR, IRS-1, and IGF-1 R were unchanged across all groups.

### Immunohistochemistry

**Figure 4:**
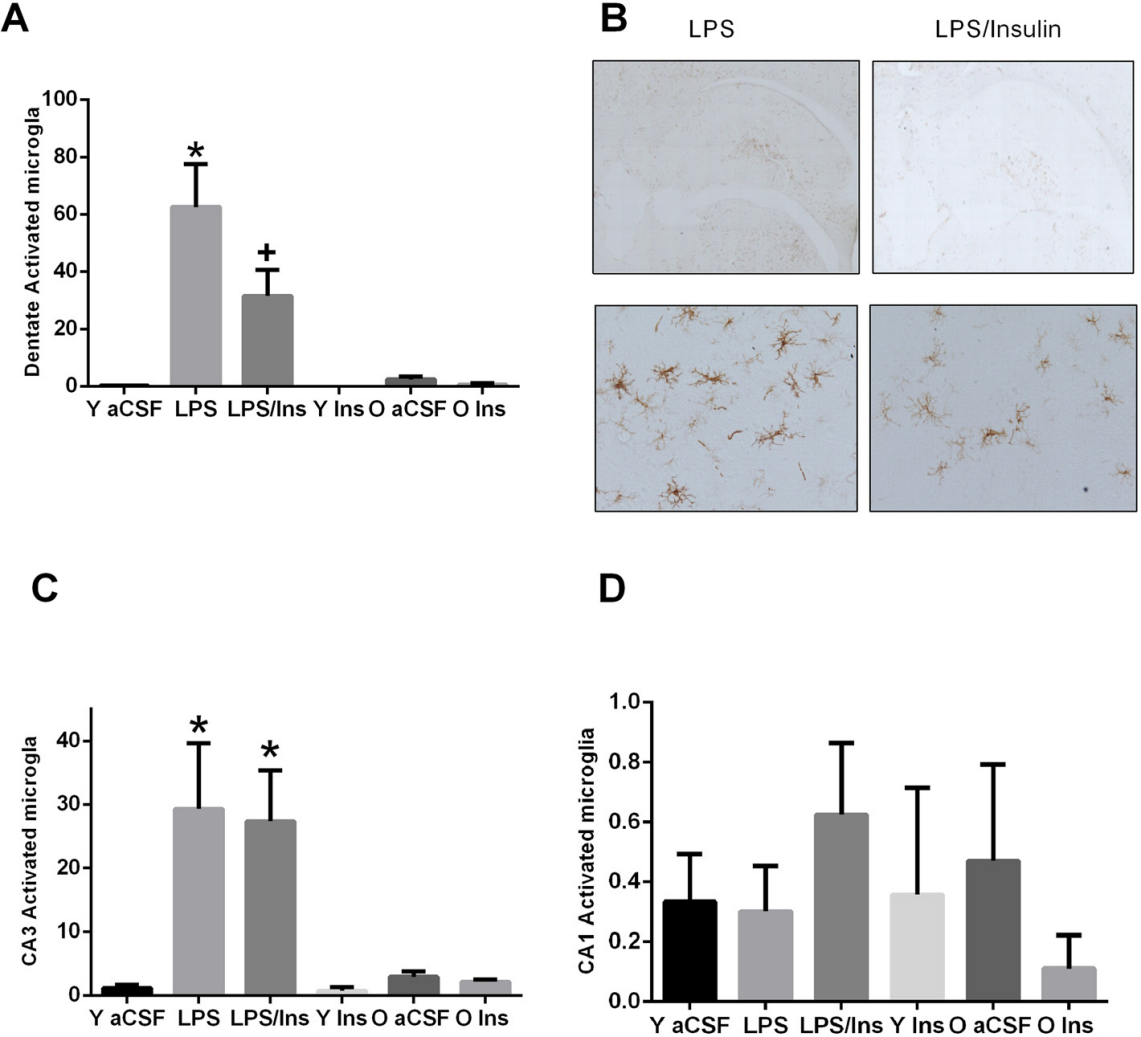
**Immunocytochemistry.** Photomicrographs and density quantification of MHC II-IR microglial in hippocampus. In the dentate gyrus **(A),** the density of MHC II-IR was significantly increased by LPS, *P* < 0.001, and significantly decreased in LPS + insulin rats, *P* < 0.05. In the CA3 region **(C),** LPS increased the density of MHC II-IR microglial cells; however, co-infusion of insulin did not decrease the density of MHC II-IR microglia. There were no observed differences in the CA1 region **(D)** between groups. There were no age-dependent changes in the density of MHC II-IR cells. **(B)** Scale bar: 400 μm for top row figures; 70 μm for bottom row. *P < 0.05 *versus* aCSF; **+** *P* < 0.05 *versus* LPS.

The increased density of MHC II-IR microglia (Figure 4) in the DG (*F*_1,81_ = 11.1, *P* < 0.001) and CA3 (*F*_1,81_ = 5.6, *P* = 0.021) regions, but not the CA1 region, of the hippocampus was significantly influenced by the LPS infusion. Insulin treatment significantly (*P* = 0.005) reduced the density of MHC II-IR microglia in the DG, but not the CA3 region. There was no significant main effect of age on the density of MHC II-IR microglia.

## Discussion

Although the precise role of insulin in the brain is not fully understood, impaired insulin signaling is a hallmark of brain aging and many neurodegenerative diseases. In peripheral tissues and cell cultures, insulin can modulate the inflammatory process by decreasing LPS-induced activation of iNOS [18] and COX-2 activity [19,51]. The role of insulin in the control of neuroinflammation remains unknown; however, recently it was observed that insulin decreased the inflammation in an *in vivo* model [52]. In the current study, we investigated effects of insulin into the brain using two unique experimental models of chronic brain inflammation: one experimentally induced by infusion of LPS; the other, naturally occurring associated with normal aging.

We have previously shown that 4 weeks of LPS infusion into the fourth ventricle of young rats induces the greatest inflammatory response that is concentrated within the hippocampus [7,46,47], a decrease in spatial, but not object recognition, memory [41], an impaired long-term potentiation [43], and an elevated levels of mRNA for multiple pro-inflammatory cytokines [41,51]. These previous findings suggest that LPS initiates a cascade of biochemical processes that show time-dependent [50,53] regional and cell-specific changes that are maximal after 4 weeks of LPS infusion. Inflammation is represented by an elevation of wide range of cytokine gene and protein expression. PKCζ protein levels, which regulates the cytokine expression [16] and NF-κB signaling [17,54], increased in response to the LPS infusion and decreased in response to the infusion of insulin leaving the phosphorylation of AKT at Threonine 308 and Serine 473 unchanged. In contrast, the PRKCζ gene, which encodes for both protein isoforms, PKCζ and PKMζ, was not affected by the LPS infusion or insulin infusion, suggesting a selective post-translational control of the production of these two proteins that are known to have unique molecular roles. The LPS infusion also increased the gene and protein expression of IL-1β as well as the mRNA level of TNFα; insulin treatment significantly decreased the expression of these neuroinflammatory biomarkers. In the periphery, insulin can reverse the effects of LPS exposure upon the levels of these markers [14]; here, we show that insulin exhibits similar actions within the hippocampus.

As previously reported [40,43,47], young rats infused with LPS demonstrated impaired performance in the MWM task, as indicated by increased latency and distance swam to find the platform, as well as increased levels of biomarkers of brain inflammation. Remarkably, all of these LPS-induced changes were significantly reversed by insulin co-infusion. This result confirms that insulin is protective against neuroinflammation in the young brain; not only did insulin reduce the impaired latency to find the platform of LPS infused young rats but also improved swim speed and the distance swam by the LPS/insulin rats compared to rats receiving only LPS. The performance of young rats in the MWM task, across all three measures, was significantly improved by insulin co-infusion. The young rats that received an infusion of insulin for 4 weeks performed significantly better than the untreated young rats; they found the hidden platform more efficiently by swimming a shorter distance at a slower swim speed.

We speculated that the specific molecular mechanism(s) underlying insulin’s ability to decrease the level of pro-inflammatory markers in the hippocampus of LPS-infused young rats and improve performance in normal young rats might be due to changes in several memory-related biomarkers such as CREB, CaMKII, PSD95, BDNF, NGF, and SYN1. However, none of these markers presented differences in their gene or protein expression. In contrast, insulin treatment did not improve performance of aged rats in the MWM task, did not alter the endogenous expression of IL-1β, TNFα, PKCζ genes, or protein levels, and did not alter the number of activated microglia within their hippocampus.

Aging is a risk factor for insulin resistance [55], and insulin signaling decrease is believed to be the cause that triggers the neurodegenerative processes [56], particularly in AD. In the current study, although IR and IGF-1R gene and protein expression was unaltered in the aged and young rats, we did see a reduced age-dependent insulin response. We also monitored blood glucose levels as well as body weight and did not find any significant changes over the 4 weeks among the different groups or ages. This result is consistent with the results of other researchers demonstrating that brain insulin does not control body weight or peripheral glucose levels [35].

## Conclusions

In summary, insulin exerts a protective function against the consequences of chronic neuroinflammation that is similar to that observed in *in vitro* peripheral tissue. Insulin administered directly into the brain can improve learning and memory in young animals but not the aged animals. Therefore, the principle role of insulin in the young brain may be to influence learning and memory and regulate the behavior of microglia; its actions may be impaired within the aged brain. Overall, these results reinforce the link between age-related insulin resistance, neuroinflammation, and cognitive impairment.

## Abbreviations

BDNF: brain-derived neurotrophic factor
CaMKII: Ca^2+^/calmodulin-dependent protein kinase II
CREB: cAMP response element-binding protein
GAPDH: glyceraldehyde-3-phosphate dehydrogenase
IL: interleukin
IL-1α: interleukin-1-alpha
iNOS: inducible nitric oxide synthase
IR: insulin receptor
IRS-1: insulin receptor substrate-1
LPS: lipopolysaccharide
MWM: Morris water maze task
NF-κB: nuclear factor kappa-B
PKB: AKT protein kinase B
PKCζ: protein kinase C-zeta
PKMζ: protein kinase M-zeta
PSD95: postsynaptic density protein 95
SYN1: synapsin I
TLR4: Toll-like receptor-4
TNF-α: tumor necrosis factor-alpha
