# Erratum to: Insulin improves memory and reduces chronic neuroinflammation in the hippocampus of young but not aged brains

**DOI:** 10.1186/s12974-015-0321-9

**Published:** 2015-08-07

**Authors:** Linda Adzovic, Ashley E Lynn, Heather M D’Angelo, Alexis M Crockett, Roxanne M Kaercher, Sarah E Royer, Sarah C Hopp, Gary L Wenk

**Affiliations:** Department of Psychology, Ohio State University, 1835 Neil Ave, Columbus, OH 43210 USA; Department of Neuroscience, Ohio State University, Columbus, OH 43210 USA

## Erratum

Following the publication of our article [1] we noticed that the western blot in Fig. 3c (Fig. 1c here) was incorrectly labelled. The S307-AKT band should instead be labelled T308-AKT. We have provided the correct figure here.

**Fig. 3:**
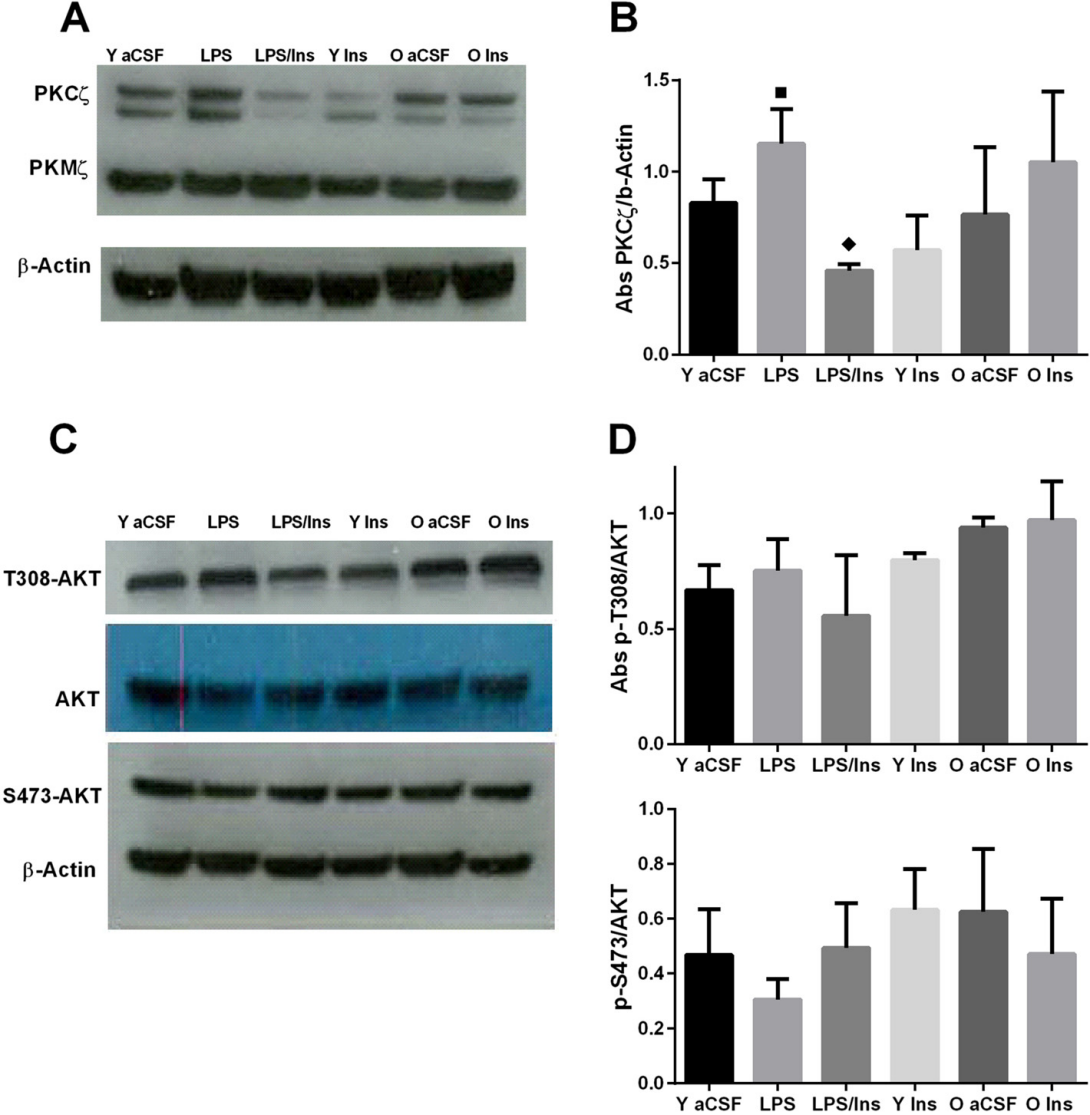
Western blot analyses. The infusion of LPS into the fourth ventricle increased the protein level (**a**, **b**) of PKCζ, black square, *P* < 0.05 *versus* aCSF. Insulin treatment reduced, black diamond, *P* < 0.001, PKCζ levels as compared to LPS. (**c**, **d**) No significant changes were observed for p-AKT Threonine 308 or Serine 473
